# Single-cell sequencing technology in skin wound healing

**DOI:** 10.1093/burnst/tkae043

**Published:** 2024-10-23

**Authors:** Xu Cheng Cheng, Wang Zi Tong, Wang Rui, Zhao Feng, Hou Shuai, Wang Zhe

**Affiliations:** Department of Pathology, Shengjing Hospital of China Medical University, Shenyang, No. 36 Sanhao Street, Shenyang 110004, China; Department of Pathology, Shengjing Hospital of China Medical University, Shenyang, No. 36 Sanhao Street, Shenyang 110004, China; Department of Pathology, Shengjing Hospital of China Medical University, Shenyang, No. 36 Sanhao Street, Shenyang 110004, China; Department of Stem Cells and Regenerative Medicine, China Medical University, No. 77 Puhe Road, Shenyang 110013, China; Department of Pathology, Shengjing Hospital of China Medical University, Shenyang, No. 36 Sanhao Street, Shenyang 110004, China; Department of Pathology, Shengjing Hospital of China Medical University, Shenyang, No. 36 Sanhao Street, Shenyang 110004, China

**Keywords:** Single-cell sequencing, Wound healing, DNA sequencing, RNA sequencing, Epigenetic sequencing, Skin, Scar, Diabetic ulcer

## Abstract

Skin wound healing is a complicated biological process that mainly occurs in response to injury, burns, or diabetic ulcers. It can also be triggered by other conditions such as dermatitis and melanoma-induced skin cancer. Delayed healing or non-healing after skin injury presents an important clinical issue; therefore, further explorations into the occurrence and development of wound healing at the cellular and molecular levels are necessary. Single-cell sequencing (SCS) is used to sequence and analyze the genetic messages of a single cell. Furthermore, SCS can accurately detect cell expression and gene sequences. The use of SCS technology has resulted in the emergence of new concepts pertaining to wound healing, making it an important tool for studying the relevant mechanisms and developing treatment strategies. This article discusses the application value of SCS technology, the effects of the latest research on skin wound healing, and the value of SCS technology in clinical applications. Using SCS to determine potential biomarkers for wound repair will serve to accelerate wound healing, reduce scar formation, optimize drug delivery, and facilitate personalized treatments.

Highlights

- Recent advancements in progressive SCS technology have ushered in a paradigm shift in the biomedical field, particularly in the realm of wound healing.
- Gene expression analysis of diverse individual cells within the periwound tissue, especially through SCS, unveils the daedal regulatory patterns at distinct wound healing stages. Incorporating SCS insights into clinical practice is conducive to facilitating personalized treatment, enhancing the accuracy of prognostic risk assessment, and optimizing drug delivery strategies.
- Comparative gene analysis between normal skin and injured tissue, by utilizing SCS, assists in identifying potential biomarkers closely associated with wound repair. This, in turn, helps in the development of novel tactics to accelerate wound healing and minimize scar formation.

## Background

According to epidemiological studies, skin wounds are mostly associated with trauma, diabetes, burns, varicose veins, the long-term bedridden, and vascular sclerosis [1, 2], where the structural integrity of the skin tissue is compromised. Specific dermatitis in inflammatory and autoimmune skin diseases can also cause wounds. If wounds do not heal within a certain period, further damage can occur, causing systemic or localized disease [3]. Furthermore, wounds can degenerate into skin cancer, such as squamous and basal cell carcinoma [4]. Factors such as the location of the damage, wound depth, size, and severity affect recovery. Hypoxia, infection, malnutrition, older age, diabetes, and obesity can result in poor wound healing, impacting all stages of the healing process [5–7]. Therefore, skin wound healing is a complex and critical research topic.

The skin damage healing process is divided into four stages: hemostasis, inflammatory response, cell repair, and wound reconstruction [8]. Additionally, a series of biological reactions is involved in the simultaneous regulation and coordination of a variety of cells, cytokines, and the extracellular matrix (ECM) [9]. With continuous research on the wound healing process, numerous cellular and molecular factors have been identified that can lead to abnormal wound healing, such as reduced expression of growth factors, decreased capillary angiogenesis, decreased macrophage numbers, abnormal structural function, and reduced fibroblast and keratinocyte growth [10]. Single-cell sequencing (SCS) is a recent development in the field of cancer genomics [11–13], providing various valuable insights into complex biological systems. SCS technology focuses on the characterization of individual cells, reveals complex and rare cell populations, tracks the developmental trajectory of different cells, and analyzes the regulatory relationships among genes [14, 15]. Considering the molecular- and cell biology-level information provided by SCS, the method has vastly improved the development of both clinical and basic medicine, providing unexpected discoveries regarding refractory wounds. This review aims to aid understanding of the mechanism and process of wound healing by discussing SCS research applications.

## Review

### SCS technology

SCS is used to obtain the protein, transcript, and gene sequence information of a cell and is followed by an in-depth analysis of the information obtained [16]. Since its discovery in 2009, SCS has been applied to numerous disciplines and fields, including neuroscience, pathogen evolution, and new-species verification. The resulting data has improved our understanding of cellular architecture and how it interacts with human, murine, and plant models [17]. SCS complements the defects of previous iterations of high-throughput sequencing, performing unbiased, high-resolution, and flux transcriptome analyses of individual cells to explore differences in gene expression and structure [3]. Therefore, SCS is an effective instrument to comprehensively analyze cellular diversity. Identification of different cell phenotypes can be used to detect wound-healing mesenchymal stem cells (MSCs), macrophage development and differentiation, and various aspects of fibroblast differentiation. SCS can also be used to study abnormal differentiation, functional keratinocytes (as well as dendritic and T cells), and other key information, thus providing novel insights into wound healing. The specific use of SCS analysis for MSCs, fibroblasts, and endothelial and epidermal cells in wound healing is discussed in the following sections.

#### Development of SCS technology

After proposal of the Human Cell Atlas project, the traditional sequencing technology (bulk-sequence) was initially developed. Traditional bulk sequencing technology cannot distinguish the differences between different cell types or subsets of cells because all of the cells are mixed together for sequencing. Additionally, traditional bulk sequencing technology cannot determine the heterogeneity of cells in tissues and provides an insufficient in-depth study on the nature of gene expression; thus, an all-round, multi-level high-throughput SCS method is necessary. Due to its high sensitivity to cell heterogeneity, SCS technology can identify rare cell subsets and novel cell types, and therefore has advantages in revealing new discoveries in the healing process of skin injuries. SCS can accurately measure the level of gene expression, trace the expression of non-coding RNA using a small sample size, providing a comprehensive and detailed perspective for understanding the healing process of skin injury. Table 1 summarizes the timeline of major SCS technologies.

**Table 1:** Timeline of major SCS technologies

**Measurement**	**Technology**	**Year**	**Reference**
Singel-cell genomic sequencing	DOP-PCR	1992	[19]
	MDA	2002	[20]
	MALBAC	2012	[21]
	Linear amplification via transposon insertion (LIANTI)	2017	[22]
Single-cell transcriptomic sequencing	Tang RNA-seq	2009	[8]
	Smart-seq	2012	[23]
	Smart-seq2	2014	[24]
	Drop-seq	2015	[25]
	CEL-seq	2012	[27]
	CEL-seq2	2016	[26]
Epigenetic sequencing	scRRBS	2013	[29]
	scATAC-seq	2015	[30]
	scDNase-seq	2015	[31]
	scChIP-seq	2015	[32]

*MDA* multiple displacement amplification, *DOP-PCR* degenerate oligonucleotide-primed polymerase chain reaction, *MALBAC* multiple annealing and looping-based amplification cycles

SCS techniques encompass the following three main types: (1) single-cell transcriptome sequencing, which involves RNA sequencing (RNA-seq); (2) single-cell genome sequencing, which involves DNA sequencing; and (3) epigenetic sequencing [18]. Each of these techniques provides unique insights into the functions and characteristics of cells at different stages, with their own developmental history and underlying principles. SCS allows for the analysis of copy number variations and point mutations at the individual cell level, enabling the exploration of relationships between cellular population differences and cell evolution. Furthermore, SCS accurately determines mutation frequencies and identifies specific mutation sources. Whole-genome amplification (WGA) is crucial for obtaining reliable DNA sequencing results but poses challenges by generating high-fidelity products. As a result, WGA methods have undergone significant advancements over time, including degenerate oligonucleotide-primed polymerase chain reaction (DOP-PCR) [19], multiple displacement amplification (MDA) [20], as well as a combination method involving MDA and PCR known as multiple annealing and looping-based amplification cycles (MALBAC) [21]. DOP-PCR uses primers with 3′ ends containing random sequences of 6 bp to enable random binding with genomic DNA for WGA. MDA uses random hexamer primers along with phi29 DNA polymerase to amplify DNA through an isothermal reaction at 30°C. Phi29 DNA polymerase exhibits high-fidelity extension capabilities for primers while demonstrating strong strand displacement activity during new chain synthesis, allowing simultaneous synthesis on both original templates and grown amplicons for rapid multiple genomic DNA amplifications. The MALBAC method is a combination of MDA and conventional PCR. In this technique, the template is annealed with a partially degenerate hybrid primer, and the amplification is catalyzed by a chain replacement enzyme. Specific primers are used to label the 3′ end of the amplification intermediates. Following one round of amplification, complementary markers are located at the 5′ end, forming loops when both ends come together. The looped amplicon is then amplified using conventional PCR. Linear amplification by transposon insertion [22] is an improved single-cell whole genome amplification method that combines transposons and primers to enable efficient sequencing. This technique not only has applications in basic research but also allows for detection of small copy number variations (CNVs) in individual cells, making it valuable for genetic screening, reproductive medicine, and understanding genetic variations in cancer and other diseases.

The use of single-cell transcriptome sequencing represents a robust approach for investigating gene expression at the individual cell level, and enables quantification of mRNA expression, functional enrichment analysis, and metabolic pathway exploration in single cells. This methodology offers numerous advantages compared to conventional gene chips, including its ability to detect subtle variations in gene expression levels, comprehend dynamic changes in gene expression patterns, and investigate disparities and similarities in transcription initiation sites. Tang *et al*. [8] pioneered single-cell RNA seq (scRNA-seq) by using reverse transcriptase to prolong the analysis time of polypeptide chains on microarrays. In their study, they introduced cDNA at the 3′ end of the polypeptide chain and used the A-tail as an anchoring mechanism. Subsequently, PCR was used to amplify the cDNA using oligonucleotide primers paired with the cDNA. This method enhances both the accuracy and efficiency in detecting gene expression changes while broadening the scope for genome detection, which is crucial for numerous vital biological processes. SMART-seq, initially reported in 2012, is a technique capable of accurately detecting full-length transcripts. Ramskold *et al*. [23] used a specialized retrovirus (the Moloney mouse leukemia virus) to extract transcriptase from Moloney mouse leukemia, which possesses terminal transferase activity and a template-switching ability. This unique enzyme facilitates the attachment of multiple free cytosines as an extension of mRNA ends. By optimizing the transcription start point coverage and enabling high-throughput sequencing of cDNA through second-generation sequencing platforms, this technique efficiently identifies gene expression differences at the single-cell level. Subsequently, researchers further refined the reaction protocol and introduced Smart-seq2 [24]. Smart-seq2 significantly enhances cDNA production and detection sensitivity for rare transcripts while eliminating purification steps and improving sequencing efficiency compared to SMART-seq. In contrast to conventional bulk RNA-seq technology, Smart-seq offers greater accuracy in identifying regulatory changes across distinct cells. Additionally, alternative protocols for transcriptome sequencing lack the ability to detect complete transcripts and instead focus on sequencing partial primers of cDNA production. These methods can be combined with cell-specific barcodes in the library to facilitate multiplexing of cDNA amplification, thereby enhancing the throughput of scRNA-seq library generation. Such approaches also allow for the incorporation of unique molecular identifiers (UMIs). For instance, Drop-seq [25] is a microdrop-based technique used for single-cell gene expression analysis. Drop-seq involves generating droplets containing a unique barcode and UMI on a specialized chip to capture individual cells. Subsequently, mRNA is merged with beads carrying these barcodes and UMIs to enable cDNA synthesis and library construction for sequencing purposes. This method ensures that each bead exclusively contains information from one cell, with each resulting cDNA labeled by a cell-specific barcode and UMI, enabling discrimination between different cell types. Moreover, Drop-seq can be used in conjunction with small molecules, mutations, and pathogens to investigate their impact on the functional response of individual cells. The perturbations induced by Drop-seq offer diverse and informative stimulus–response data at the multicellular level. The CEL-seq2 [26] method enhances the original CEL-seq [27] technique for single-cell transcriptome sequencing on microfluidic chips. CEL-seq2 differs from Drop-seq in terms of DNA labeling, the addition of UMIs, and reverse transcription. Additionally, CEL-seq2 uses *in vitro* linear amplification instead of PCR amplification. The amplified barcode RNA is collected and segmented from the chip, followed by reverse transcription to obtain sequencing libraries. Among these scRNA-seq methods, Drop-seq is well-suited for quantifying the transcriptome of a large number of cells, whereas SCRB-seq and MARS-seq are suitable for low sequencing depth quantification. For annotating or counting the transcriptome of a small number of cells, Smart-seq2 is the preferable choice [28].

Cellular heterogeneity is predominantly determined by epigenetic heterogeneity. The transcriptional expression of genes is influenced by the chromatin state, encompassing factors such as transcription factor binding, exposure to *cis*-acting elements (e.g. enhancers and promoters), and the impact of 3D conformation and histone modifications on both chromatin state and transcriptional burst randomness. Correspondingly, diverse SCS techniques have been developed at various epigenetic levels. The development of single-cell reduced representation bisulfite sequencing (scRRBS) has facilitated the analysis of methylated groups at the individual cell level. Guo *et al*. [29] enhanced the conventional RRBS protocol by successfully integrating all reaction steps into a single tube, thereby minimizing DNA loss during repeated purification. An average of 40% of the murine genome CpG islands were detected in single cells, thus enabling exploration of dynamic changes in DNA methylation within individual cells. Chromatin accessibility refers to the degree to which nuclear macromolecules physically interact with chromatin DNA, influenced by nucleosome occupancy and topology, as well as other chromatin binding factors that hinder access to DNA. The singel-cell Assay for Transposase Accessible Chromatin with high-throughput sequencing (scATAC-seq) [30] and scDNase-seq [31] techniques were used to conduct single-cell chromatin accessibility assays. The genomic regions targeted by DNase in scDNase-seq and the Tn5 transposase in scATAC-seq are referred to as DNase hypersensitive sites. This is because the Tn5 enzyme fragments the genome and adds adapter sequences simultaneously within a single cell, enabling high-throughput detection in scATAC-seq. In DNA sequence analysis, DNases also fragment DNA within open chromatin regions. However, due to the subsequent addition of adapters during library preparation, the throughput of this method is lower compared to that of scATAC-seq. The singel-cell chromatin immune-precipitation (scChIP-seq) technique [32] is used to investigate protein–DNA interactions. The enhanced ChIP-seq approach integrates traditional ChIP-seq with other methodologies, such as Drop-ChIP, to enable the detection of chromatin modifications at a single-cell level, facilitating improved analysis of chromosomal cross-linking data. This technique can be applied not only to the examination of histone modification but also to exploring the interaction between DNA and non-histone proteins, such as transcription factors.

#### SCS application directions

The SCS technique can be divided into numerous stages, including single-cell isolation, DNA amplification, library building, high-throughput sequencing, and data analysis. The most important step is obtaining target cells with high vitality. These methods include the gradient dilution method [6], laser capture microscope cutting [33], microfluidic technology [34], and fluorescence-activated cell sorting (FACS) [35]. The arrival of single-cell isolation technologies, as well as next-generation sequencing platforms, such as the BD Rhapsody™ Single-Cell Analysis System [36] and 10X Chromium Single Cell Gene Expression Solution [37] SCS technology, have become increasingly advanced and their use has been expanding.

Currently, droplet-based 10X Genomics and SMART-seq are two commonly used platforms that have their own advantages and disadvantages. However, the droplet-based 10X Genomics Chromium can only detect the 3′ or 5′ end of a biased transcript, and requires a large number of cells in a single sample (> 90% recommended). The 10X Genomics Chromium also has a lower cell-capture rate than SMART-seq, which makes it unsuitable for detecting rare samples with small numbers of cells [38]. SMART-seq v2 and v4 [39] are now widely used in cancer research [40], require no additional equipment, and do not require experienced researchers. A recent study [41] compared the features of 10X Genomics and SMART-seq by directly comparing scRNA-seq data generated from the same CD45 cell samples using an extensive analytical system. While SMART-seq is sensitive, 10X Genomics can sequence more cells in a single run and is more cost-effective than SMART-seq. At present, 10X Genomics technology is the mainstream choice. The Illumina HumanMethylation450 BeadChip, epigenome-wide association studies, International Cancer Genome Consortium, and International Human Epigenome Consortium have become popular platforms for examining DNA methylation [42].

Currently, when SCS is widely used, its generated high-dimensional datasets also present special analytical challenges [43]; various bioinformatics tools have been developed to analyze these datasets [44]. When SCS is complete, processing the original sequencing data is necessary to acquire the genetic expression information for each cell. This comprises qualitative evaluation of sequencing reads, alignment with a reference genome, division of a cell bar code, and quantitation of reads or transcripts per gene [45]. Gene expression may change along the variable axes so that differential expression occurs among the clusters. Trajectory analysis can reveal deep biological relationships by determining the type and status of the cell by allocating directionality to every cell in a dataset [46]. Additionally, the single-cell regulatory network inference and clustering (SCENIC) method, which relies on gene expression [47, 48], can reveal the dynamic role of various genes in single cells, construct a framework of gene connection and regulation, and reveal regulatory changes responsible for different clinical manifestations.

### Applications of SCS in wound healing

Wound repair requires multiple cell types, including keratinocytes, fibroblasts, vascular endothelial cells, and immune cells. These cells work together to restore skin barrier function [10]. For example, fibroblasts play an important role in wound healing, especially promoting the deposition of ECM mediated by fibrotic growth factor. Fibroblasts from different tissues, different developmental stages, and different states have obvious heterogeneity, which leads to significant phenotypic differences and thus translates into different functions in wound healing [49]. Moreover, macrophages can secrete a variety of chemokines and growth factors that play an important role in cell proliferation, angiogenesis, and the formation process of wound healing [50]. In addition, vascular endothelial cells are not only involved in all stages of normal wound healing, but also act as sensors and effectors to translate complex local mechanics into cellular responses and biochemical signals. Therefore, endothelial cell dysfunction may play an important role in wound healing [51]. Through tools such as SCS, researchers can gain a higher understanding of cells from different lineages, and a full understanding of their roles will be beneficial to elucidate the mechanisms of wound healing.

#### scRNA-seq applications

**Figure 1:**
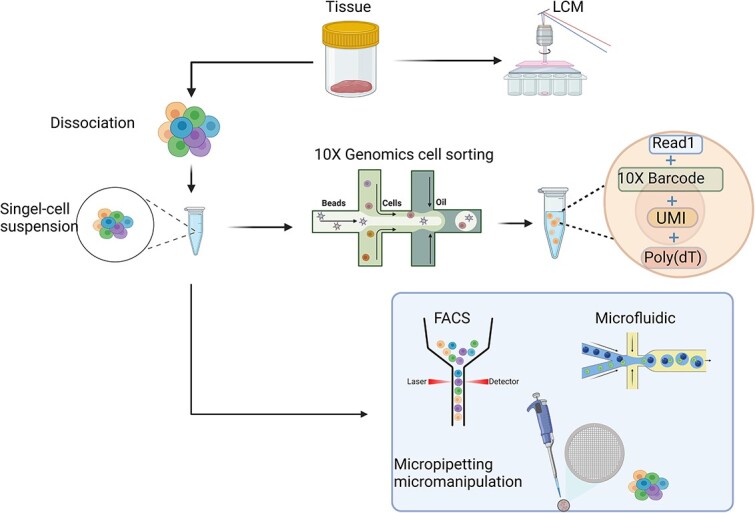
Different approaches to isolate and capture single cells for single-cell RNA sequencing. Cells are freed from the extracellular matrix and cell–cell adhesion (except for circulating cells), which is often achieved via enzymatic treatment, but can also be accomplished by laser capture microscope cutting and patch clamping. Once a single-cell suspension is obtained, several methods are used to deliver cells into individual reaction tubes. Micro pipetting (manual or automated) is often used to capture cells from samples that do not contain many cells. The 10X Genomics platform uses advanced microfluidic technology to enable high-throughput analysis of the 3′ end of single-cell RNA with exceptional capture efficiency. The platform uses microdroplet microfluidics to achieve single-cell separation, enabling the monodispersion of water droplets within a continuous oil phase. The sample cells are then introduced and immobilized onto the gel beads at the initial intersection and undergo oil-droplet coating at the subsequent intersection, thereby achieving single-cell capture. *LCM* laser capture microscope, *UMI* unique molecular identifiers

RNA-seq is a powerful tool that is used to assess cell-to-cell differences, facilitate identification of cell populations, and clarify cellular heterogeneity [52]. The transcriptome is the sum of all transcribed RNA, including mRNA and non-coding RNA, in a certain developmental stage and function [53, 54]. Therefore, RNA-seq, i.e. transcriptome sequencing, is essential for the analysis of differences in the expression of mRNA and protein assembly [55]. The different methods for isolating and capturing single cells for scRNA-seq are shown in Figure 1.

##### scRNA-seq applications for MSCs

In recent years, the function of MSCs, their multilineage differentiation, immune regulation, and self-renewal for wound healing have been the central foundation for the process of skin healing because their concentration in the wound activates the autophagy of other cells, accelerates fibroblast division, and accelerates complete wound healing [56]. MSCs are highly favored therapeutic agents that present regenerative potential. Variations occur in the heterogeneous expression profiles of MSCs obtained for therapeutic purposes, which can be attributed to factors such as age, sex, tissue origin, and isolation conditions. Furthermore, identifying specific surface markers and their functions is crucial to comprehend MSC differentiation in regenerative medicine. SCS technology enables transcriptome and regulator analysis involved in MSC differentiation, thereby enhancing prediction and identification of MSC differentiation trajectories.

In studies using human tissue-level sources, MSCs such as adipose-derived stem cells (ADSCs) show great promise in clinical applications, as they are more accessible than embryonic stem cells, with no ethical problems. ADSCs are promising pluripotent stem cells that have been developed for repair and regeneration of tissues, such as soft tissue recovery and skin healing [57]. Wang *et al*. [58] explored the influence of exosomes (Exos) from ADSCs on the potential mechanism of wound healing in mice, and the Exos from ADSCs and fibroblasts were sequenced for RNA. The results showed that the Exos of ADSCs expressed more circ-Astn1 than those of fibroblasts. Exos with high concentrations of circ-Astn1 promote silent information regulator sirtuin 1 (SIRT1) expression, thereby increasing the therapeutic effect of endothelial progenitor cells (EPC) function in hyperglycemic conditions. ADSC-Exos provide a potential therapeutic avenue for the treatment of wound healing in patients with diabetes. To investigate the inter-population heterogeneity of primary cultured Wharton’s jelly MSCs (WJMSCs) at the single-cell transcriptomic level, Sun *et al*. [59] collected primary cells from three human umbilical cords and analyzed their gene expression profiles using scRNA-seq. Gene ontology enrichment analysis and pathway analysis revealed that highly variable genes in WJMSCs were associated with the functional characteristics of classical MSCs. Differential expression gene analysis identified several distinct subgroups, which were further separated by flow cytometry into CD142+ and CD142− WJMSCs. CD142 (F3) was expressed the highest in subgroup C3 and least in subgroup C0. By comparing the single-cell transcriptome data of WJMSCs and adipose tissue-derived mesenchymal cells (ADMSCs), shared and highly expressed classical MSC surface markers were found between the two cell types, as well as some unshared differentiation clusters, indicating phenotypic diversity between WJMSCS and ADMSCs. Finally, calculating lineage differentiation scores using single-cell gene expression data from WJMSCs and ADMSCs demonstrated a tendency for osteoblastic lineage differentiation in WJMSCS, whereas ADMSCS tended to differentiate into adipocytes. Lin *et al*. [60] discovered that SCS technology could track and analyze transcriptomic changes during MSC differentiation. Moreover, an increase in *in vitro* heterogeneity was observed with cell passage number, revealing the multidirectional differentiation potential and trajectory of these cells.

In studies using animal tissues and cells, Schwalie *et al.* [61] used scRNA-seq to investigate the process of adipogenesis in mammalian fat depots and identified stromal cell populations that inhibit this process. The research team performed scRNA-seq on Lin− cells from the subcutaneous stromal vascular fraction of mice using the 10X Genomics Chromium platform, analyzing a large number of cells to identify stromal cell populations associated with adipogenesis and further elucidate their function and regulatory machinery. Through functional follow-up experiments on these stromal cell populations and gene knockout studies, the researchers identified a subset of cells, adipogenesis regulators, that suppress adipogenesis by secreting specific proteins that inhibit adipocyte differentiation and proliferation. Merrick *et al*. [62] used scRNA-seq and the 10X Genomics platform to isolate and analyze subcutaneous groin cells from both mice and humans. Using CD45 gating and FACS sorting techniques to exclude non-target cells, the GemCode instrument, and Cell Ranger single-cell software for sequencing and analysis, three distinct sets of MSCs were identified in mice adipose tissue: (1) DPP4+ mesenchymal progenitor cells, (2) ICAM1+ preadipocytes, and (3) CD142+/Clec11a + cells. This study systematically elucidated the hierarchy of intermediate mesenchymal progenitor cells within adipose tissue for the first time, revealing their distribution, function, heterogeneity, and diversity, thereby providing novel insights into further investigations on the physiological and pathological processes associated with adipose tissue. Moreover, adipose tissue offers a new target and strategy for combating obesity-related diseases. The high level of agreement observed across studies underscores the robustness of the scRNA-seq technique. These findings highlight the critical role played by stromal cell populations during adipogenesis and reveal a novel mechanism for inhibiting this process, which has important implications for understanding obesity-related metabolic disorders as well as developing new therapeutic strategies for wound healing. These experiments showed that MSCs participate in angiogenesis, cell migration, cell growth, and morphogenesis to accelerate cell migration and promote wound healing. Gaining an extensive understanding of individual and population differences among MSCs will enhance our comprehension of their biological complexity and support various applications in wound healing, laying the foundation for subsequent clinical treatment and research on wound healing.

##### scRNA-seq applications for fibroblasts

Fibroblasts, as the principal dermal parenchymal cells, play a pivotal role in maintaining skin homeostasis and facilitating tissue repair. Fibroblasts serve as crucial effector cells during wound healing [63]. New discoveries have been reported regarding fibroblasts, such as subgroups, differences in subgroup composition and communication between fibroblasts and other cell types, and new therapeutic targets. In addition, the new and powerful SCS technologies have enabled an extensive understanding of fibroblast biology and fibroblast-mediated pathology, based on an improved understanding of the functional diversity of fibroblast phenotypes in healthy and pathological states [64].

Fibroblasts originate from MSCs in the embryonic mesoderm and are the most common cell type in connective tissue. MSCs are fibroblast-like pluripotent stem cells with multiple differentiation potentials and are the most common cell type used in cell therapy and tissue repair [65]. In wound healing, the MSC source is donor-friendly, and the obtained cells have higher proliferation and differentiation ability, which will also affect the effectiveness of fibroblasts in wound treatment [66]. There are two methods to induce MSCs to differentiate into fibroblasts. One is to add cytokines or co-culture with other cells to promote the differentiation of MSCs into fibroblasts. The other is to construct related stereoscopic scaffolds to induce differentiation. Thus, the two types of cells are largely similar, with differences mainly in morphology and related terminology. ADMSCs and fibroblasts do not have detectable levels of telomerase activity, and their proliferative capacity is similar. In terms of differentiation ability, osteogenic, lipogenic, and myogenic differentiation of fibroblasts have been observed. As pluripotent stem cells, MSCs can also differentiate into chondrocytes, adipocytes, and osteoblasts *in vitro*. In terms of immunomodulatory properties, MSCs demonstrate major histocompatibility complex Class I (MHC class I) expression, leading to non-recognition by natural killer cells, but no MHC class II expression, leading to non-recognition by alloreactive CD4+ T cells. The degree of inhibition of T cell proliferation by dermal fibroblasts is similar to that of MSCs, and the fact that MHC class II is not expressed by fibroblasts is also conducive to their not being recognized by T cells [67]. In general, because of the related characteristics of the two, they are mostly used in clinical wound healing, skin wounds, inflammation suppression etc..

Fibroblasts have been implicated in various biological processes, including wound healing, ECM remodeling, immunity, and stem cell differentiation [68]. After the appearance of the wound surface, the fibroblasts and myofibroblasts around the wound are stimulated to proliferate. Fibroblasts and myofibroblasts are drawn to elements such as transforming growth factor (TGF) and platelet-derived growth factor (PDGF) released from inflammatory cells and platelets, and migrate to the wound [69]. In studies using human tissue-level sources, diabetes, among other chronic diseases, are characterized by localized and systemic low levels of inflammation, and a hyperglycemic environment severely compromises the migratory capacity of keratinocytes [70]. According to Theocharidis *et al*. [71], mRNA-seq was performed for a group of cured and incurable chronic diabetic foot ulcers to analyze cells near the wound surface, and the results showed multiple polyfibroblast clusters and increased inflammation. Subsequently, by substantially increasing the sample size [72], comprehensive mapping of the diabetic wound healing system using bulk mRNA-seq revealed the presence of fibroblasts near the wound surface. These cells were enriched in ECM- and inflammation-related genes, including matrix metallopeptidase 1, metallopeptidase 3, and interleukin 6, as confirmed by SCS and immunostaining. These findings lay the foundation for wound healing in diabetes and describe the multifaceted roles of fibroblasts in wound healing, indicating the potential of fibroblasts as therapeutic targets.

Guerrero-Juarez *et al*. [73] used RNA-seq to analyze cells in murine skin wound tissue. Upon completion of re-epithelialization on day 12, large wounds may contain two major populations of fibroblasts, with one population comprising 24% of wound fibroblasts and consisting of three subpopulations (sC3/9/C11), and a second, heterogeneous population comprising 76% of wound fibroblasts and consisting of nine subclusters. The researchers also identified different cell types in the wound tissue, including fibroblasts, bone marrow-derived fat cell progenitors, and other immune cells. This was followed by the discovery by RNA-seq that many fibroblasts expressed hematopoietic markers, which then demonstrated a population of myofibroblasts with myeloid characteristics that persist in the wound during repair and regeneration, and may result in the dynamic changes of these cells during wound healing through myeloid cell reprogramming. In addition, animal models showed heterogeneity in wound-induced skin regeneration, establishing that bone marrow-derived adipocytes and a rare subpopulation of injured fibroblasts with bone marrow characteristics were involved in adipocyte regeneration. RNA-seq was used to gain insight into the heterogeneity of different cell types and the origin of adipocyte progenitor cells in murine skin wounds. This allows a comprehensive understanding of cellular interactions and regulatory mechanisms during wound healing, providing a theoretical basis for developing new therapies and promoting wound healing. These findings are expected to promote the effectiveness and speed of wound healing. Chen *et al*. [74] found that although fibrous scars are common in both human and murine skin wounds, trauma-induced hair regeneration in murine trauma models usually leads to a regenerative repair response. Therefore, single-cell RNA-seq was performed on dermal cells from murine skin wounds on Day 18, and two different healing outcomes were found, namely fibrosis or regeneration. The 2000 most variable genes in dermal cells were then analyzed using the data from SCS and six cells were annotated, including myofibroblasts and macrophages. Finally, the heterogeneity of myoblasts or macrophages may determine the fate of wound healing, whether via regeneration or fibrosis. Sun *et al*. [75] established a murine model of full-layer skin defect wounds and performed single-cell RNA-seq on both wound tissue and normal skin tissue at 7 days post-injury. Consequentially, a gene expression matrix was obtained for different cell populations and used to investigate the intercellular communication network during skin wound repair. The dermal fibroblasts (dFbs) group was especially monitored among the different cell populations. The dFb subgroups, C0, C1, and C2, exhibited the highest levels of communication intensity, with communication links observed between these three dFb subgroups and other cell populations within the wound tissue. The study also identified that intercellular communication relied on a signal network involving multiple growth factor interactions, particularly focusing on potential targets such as vascular endothelial growth factor, PDGF, epidermal growth factor, and fibroblast growth factor, which participate in wound repair. Combining these findings with scRNA results indicated that dFb acted as both sender and receiver within multiple growth factor networks. Different dFb subgroups played distinct roles in various growth factor signaling pathways, further highlighting their high heterogeneity and central position during wound repair processes. These discoveries contribute to our understanding of cellular interactions during wound healing and provide a theoretical foundation for developing growth factor-related therapies for efficient wound repair. Muhl *et al*. [76] primarily investigated the heterogeneity of fibroblasts and mural cells, as well as the criteria for their identification and differentiation. Transcriptome analysis of individual cells in four murine muscle organs (heart, skeletal muscle, intestine, and bladder) yielded a comprehensive catalog of organ-specific markers for fibroblasts and parietal cells on a genome-wide level. These results revealed distinct gene expression signatures that discriminate between fibroblasts and parietal cells, providing valuable molecular markers for cell subtype identification. The transcriptome data also unveiled shared markers defining fibroblasts across all organs, while highlighting significant interorgan heterogeneity within the stromal compartment in terms of gene expression patterns. In contrast to fibroblasts, vascular wall cells exhibited limited cross-organ heterogeneity. Smooth muscle cells (SMCs) displayed minimal variation except for two specific SMC populations: colonic interstitial SMCs and those originating from large blood vessels connected to the heart. The characterization of different subtypes of fibroblasts and parietal cells not only reveals intra-organ diversity but also highlights common features across multiple organs. These findings will contribute to understanding the roles played by these cell types in wound healing processes.

##### scRNA-seq applications for endothelial and epidermal cells

Endothelial cells, which form the inner walls of blood vessels, can swallow foreign bodies. In addition, endothelial cells participate in immune system development to facilitate the development of new vasculature and actively contribute to wound healing. In studies originating from human tissue-level sources, keloids are observed to be a consequence of wound healing disorders; therefore, endothelial dysfunction may play an important role in the development of keloids. Shim *et al*. [77] conducted SCS and spatial transcription analysis on keloid tissue samples and normal skin tissues and revealed the heterogeneity of endothelial cells, fibroblasts, and other cells by cRNA-seq. To assess the heterogeneity of fibroblasts in keloids, a total of 11 562 fibroblasts were collected, and multiple distinct populations were identified in both keloid and normal skin samples. Notably, fibroblast1 (FB1) and fibroblast2 (FB2) clusters comprised 90.5% of the keloid lesion cells and exhibited significant upregulation of previously established keloid marker genes. Subsequently, spatial transcription analysis conducted on sections from normal skin and keloids revealed that regions with elevated keloid scores were predominantly localized within deep lesions, particularly surrounding areas exhibiting endothelial transcripts. Compared with normal cells, activation of the mesenchymal of keloid endothelial cells, characterized by dysregulation of the transforming growth factor-beta1 (TGF-β)/Smad signaling pathway, promotes neovascularity leading to keloid formation and abnormal fibrosis. Thus, the potential action of fibrovascular communication and mesenchymal activation of endothelial cells on the pathogenesis of keloids is demonstrated. This information provides new insights into the molecular basis of keloid pathogenesis.

In data from animal tissue-level sources, murine epidermis and hair follicles represent a valuable model of tissue regeneration. The results of scRNA-seq using a 10X Genomics-based single-tube protocol for single-cell mRNA transcriptome studies from 11 different murine tissues [78] explored the endothelial cells of different vascular beds in different tissues, showing heterogeneity of metabolic transcriptomic characteristics, and found that the heterogeneity of endothelial cells to be apparent in different tissues. Joost *et al*. [79] performed scRNA-seq to uncover the underlying regulatory mechanism for the heterogeneities in murine epidermis cells at the transcription level. A total of 25 distinct cell types were identified from 1422 individual cells that were transcribed and grouped according to their signature genes. For example, sebaceous gland cells are characterized by stearoyl-coenzyme A desaturase 1/microsomal glutathione S-transferase 1. Langerhans and T cells are characterized by cluster of differentiation 207 (CD207) and CD3, and the indicators of keratinocytes are keratin 6a/keratin 75 and CD34. A systematic view of the transcriptional organization in the epidermis using scRNA-seq, which allows for a variety of murine epithelium components to be sequenced, offers key insights into both normal and wound healing conditions, emphasizing the coordination of cellular diversity in the body to maintain tissue equilibrium. Haensel *et al*.’s [80] study on transcription and metabolism heterogeneity between follicular epidermis base cells in healthy and injured skin further explored the coordination of cell heterogeneity *in vivo* and analyzed basal cell dynamics during differentiation. Stem and precursor cells in the base layer of adult mice were able to regenerate themselves or undergo mitotic differentiation, ultimately creating an extracellular permeable barrier to protect the skin against water loss or other damage and promote wound healing. Using scRNA-seq, one proliferative and three non-proliferative basal cell states were identified; these states altered gene expression during wound healing. By combining RNA *in situ* detection (RNAScope) and fluorescence lifetime imaging microscopy, the molecular and metabolic heterogeneity revealed via RNA-seq was localized to intact normal or injured murine skin tissues, demonstrating enhanced cell fate and fluidity during wound healing. This study investigated epidermal cell dynamics in the normal steady and repair states from a single-cell perspective, providing insights into the mechanisms of skin maintenance and repair.

##### Single-nuclear RNA-seq

Single-nuclear RNA sequencing (snRNA-seq) is a relatively new and powerful technique [81] for transcriptome sequencing of a single-cell nucleus, a revision or alternative to scRNA technology. Currently, snRNA-seq is mostly used for SCS of hard-to-isolate tissues such as the brain and kidneys [82, 83]. Gene sensitivity detected by the two sequencing methods is similar. ScRNA-seq can detect RNA isolated from cells in tissues, while snRNA-seq can analyze frozen or hard-to-isolate tissues [84]. Most exons are read by scRNAseq, and introns are read by snRNA-seq. Relatively speaking, although the proportion of snRNA-seq in different genes is lower than that of scRNA-seq, snRNA-seq can detect cell types not detected by scRNA-seq, which better reflects the cell types and gene expression in the original tissue and avoids the influence of abnormal transcription to the greatest extent. This is a major advantage in understanding complex groups of organizations [85]. SnRNA-seq, which has neither the need for fresh specimens nor the limitations of dissociation-induced cell loss and deficiency, may be a promising approach for wound skin biology. Zhu *et al*. [86] conducted snRNA-seq and scRNA-seq on human bisected skin and analyzed the differentiation tracks of keratinocytes and fibroblasts, in particular by analyzing the differences in gene expression and cell proportion between the two. The results showed that fibroblasts were activated in snRNA-seq. LY6/PLAUR Domain Containing 3 (LYPD3) and Cystatin-B (CSTB) are new markers of different differentiation stages of keratinocytes. Sun *et al*. [87] collected adipose tissue samples from mice and humans and sequenced these samples successively using snRNA-seq technology to identify and study the adipose cell subpopulations that regulate heat-energy production. Three cell subpopulations were first identified in mice, nuclei preserved under three different temperature conditions were analyzed, and 10 subpopulations were found, of which the P4 subgroup included three temperature conditions. Next, snRNA-seq of human fat cells tested positive for several subpopulations of P4 markers compared to mice. To further characterize P4 cells, the expression of the most significant marker gene, Cyp2e1, was analyzed to verify and further explore the function and mechanism of these adipocyte subsets. Through follow-up experiments and analysis, the research team successfully revealed a subpopulation of fat cells that regulates heat energy production and conducted in-depth studies on its role in thermoregulation. The results of this study have important implications for understanding the function of fat cells and the mechanisms of heat-energy regulation.

In fact, snRNA-seq is less susceptible to contamination by mitochondrial and ribosomal RNA and shows stronger transcription factor detection capabilities than scRNA-seq. The two tests complement each other and update the skin transcriptome map, laying a foundation for further exploration in the field of wound healing in the future.

#### DNA sequencing applications

**Figure 2:**
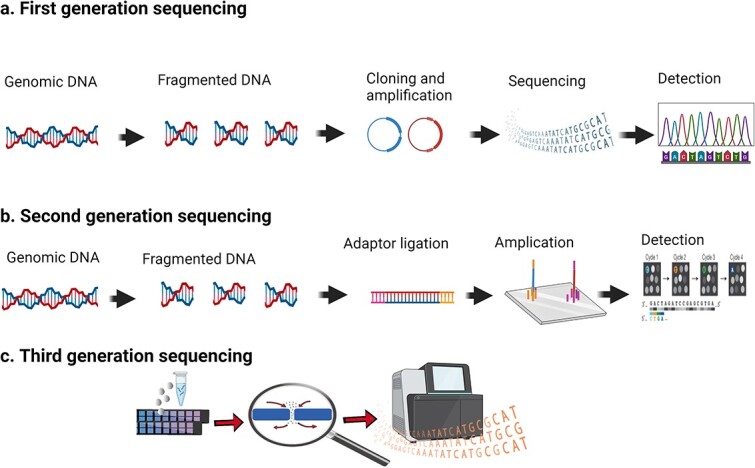
DNA sequencing technologies. (**a**) First generation sequencing. Using high-throughput shotgun Sanger sequencing, genomic DNA is fragmented and cloned into a plasmid vector. For each sequencing reaction, a single bacterial colony is selected, and plasmid DNA is isolated. As fluorescently labelled fragments of discrete sizes pass through a detector, a four-channel emission spectrum is used to generate a sequencing trace. (**b**) Second generation sequencing. In shotgun sequencing with cyclic-array methods, common adaptors are ligated to fragmented genomic DNA, which is then subjected to one of several protocols that results in an array of millions of spatially immobilized PCR colonies or ‘polonies.’ Similarly, imaging-based detection of fluorescent labels incorporated with each extension can be used to acquire sequencing data for all features in parallel. Successive iterations of enzymatic interrogation and imaging are performed to construct contiguous sequencing reads for each array feature. (**c**) Third generation sequencing. Nanopore single-molecule sequencing technology is a third generation sequencing technology. Compared to the previous two generations, their greatest feature is single-molecule sequencing, which does not require PCR

Over time, DNA sequencing technology—now in its third generation—has developed significantly, with each generation presenting its own advantages. With the continuous emergence of novel technology, the number of clinical applications is increasing [88]. Single-cell DNA sequencing has proven to be more challenging than RNA-seq, since RNAs are abundant in every cell; however, but there are only two copies of DNA [89]. The gross model of three-generation DNA sequencing is shown in Figure 2. In this paragraph the diagnosis, treatment, and development of wound healing at the DNA sequencing level is discussed.

Human skin resembles an independent ecosystem in which there are different kinds of bacteria, viruses, and fungi, and despite long-term exposure to the external environment, bacteria and fungi are stable for a period of time. The skin microbiota are essential to a wide range of bodily functions [90]. Oh *et al*. [91] conducted a metagenomics study of skin samples from 17 body parts of 12 volunteers at three time points between 2011–2014 and found that most of the microorganisms were relatively stable; however, the relative abundance of a few species, such as *Propionibacterium acnes* and *Staphylococcus epidermidis*, varied significantly over time. Infection-induced sepsis is a fatal complication for patients with burns. Feng *et al*. [92] studied skin and blood samples from burn patients and patients with acute and chronic wound infections, sequencing randomly selected nucleic acid fragments from extracted nucleic acid samples. Non-concurrent fractures may fail to heal due to various reasons, and the potential presence of infection is challenging to detect using conventional methods. Goswami *et al*. [93] used next-generation sequencing (NGS) technology for microbiota detection and identification. Through a prospective multicenter cohort study involving 37 patients undergoing open fracture surgery and 17 controls with acute fractures, they collected samples and applied NGS technology. The effects of treatment were monitored and compared with those who achieved healing to evaluate the association between persistent or emerging infections and non-healing conditions. The study revealed that NGS identified microorganisms in a significant proportion of culture-negative cases of bone nonunion. Moreover, at the DNA level, some instances of nonhealing may involve multiple microbial species that cannot be detected by traditional culture methods. Therefore, NGS serves as a valuable adjunct for identifying nonhealing organisms in an environment where cultures yield negative results, thereby laying the groundwork for treating wound nonhealing caused by the microbiome. Diabetic foot infection is a skin and soft tissue infection caused by a high-glucose environment that may lead to the continuous spread of microorganisms through the wound surface to the bone structure, resulting in diabetic foot osteomyelitis [94]. Malone *et al*. [95] found that DNA sequencing is more sensitive than conventional cultures for detecting infectious pathogens in samples from patients with diabetic foot osteomyelitis. Pathogenic bacteria can be detected sensitively using DNA sequencing, which provides strong evidence for the detection of microorganisms in burns, orthopedic trauma, and acute and chronic infections, and provides auxiliary benefits for the healing of various refractory wounds.

#### Epigenetic sequencing applications

**Figure 3:**
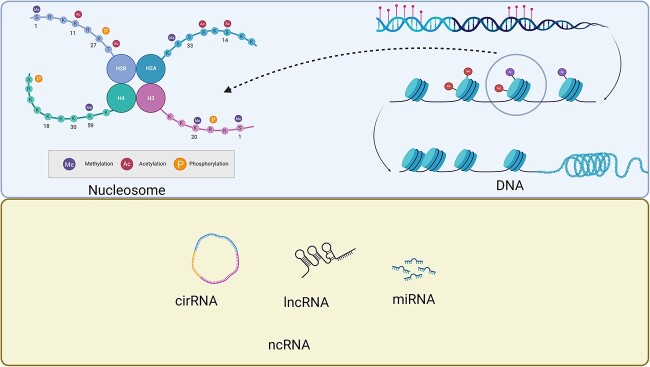
Epigenetic regulation landscape in wound healing. Epigenetic modifications in wound healing include DNA methylation, histone modifications, and non-coding RNAs (ncRNAs). DNA and histone protein modifications, including methylation (Me) and acetylation (Ac), can regulate the transcription process by affecting chromatin looseness. DNA methylation refers to the transfer of a methyl group to the C5 position of cytosine in CpG dinucleotides, which confers additional epigenetic regulation in the cytoplasm. MicroRNA (miRNA), long non-coding RNA (lncRNA), and circular RNA (circRNA) are the major ncRNA types involved in wound healing

Epigenetics refers to the altered gene expression caused by non-genomic sequence changes, including non-coding RNA regulation [96], chromosomal remodeling [97], DNA methylation [98, 99], and histone modifications [100]. Epigenetics mostly regulates gene translation and transcription and has an impact on their characteristics and functions [101]. This could provide new insights for treating wounds. The genetic landscape for the regulation of wound healing is shown in Figure 3.

To investigate the regenerative capacity of skin fibroblasts during wound healing and study its regulatory mechanism, Abbasi *et al*. [102] used single-cell transcriptomics and single-cell ATAC-seq techniques to further explore the function and properties of skin fibroblasts. First, scRNA-seq was conducted on 29 269 single cells from small wounds (SW) at 8 and 14 days post-wounding (DPW), as well as large wounds (LW) at 14 DPW in undamaged skin, aiming to analyze the molecular programs that determine the fate of different fibroblast populations during fibrosis and regenerative healing. The analysis using a droplet-based 10X Genomics system revealed significant overlap in gene expression between LW periphery (LWP) fibroblasts and LW center (LWC) fibroblasts, suggesting that regionalized fibroblast plasticity may be influenced by post-transcriptional or epigenetic mechanisms. Subsequently, single-cell ATAC-seq was performed to assess genome-wide chromatin accessibility specifically in LWC dermal fibroblasts at 14 DPW. Among the identified differential enrichments across 579 motifs, numerous transcription factors were collectively predicted to exhibit differential accessibility and activity in upper and lower dermal fibroblasts. Finally, integration of scATAC-seq with sc-RNA-seq data in LWC dermal fibroblasts at 14 DPW unveiled complementary regulatory control layers governing their function while highlighting key transcription factors that could serve as crucial molecular targets for promoting successful skin regeneration or alleviating fibrosis. These findings have significant implications for understanding the mechanisms underlying skin regeneration and wound healing processes, while also providing potential targets for developing novel treatments and drugs. Foster *et al*. [103] used a murine model to achieve stochastic lineage tracking and clonal analysis of fibroblasts. The study used a combination of scRNA-seq and scATAC-seq techniques to scrutinize fibroblasts at distinct temporal points and spatial locations. By integrating these two datasets, they leveraged the ArchR platform to investigate the interplay between chromatin accessibility and gene expression, thereby uncovering alterations in gene expression and chromatin accessibility that drive wound closure and fibrosis. Furthermore, batch RNA-seq data was subjected to analysis using the CIBERSORTx deconvolution algorithm to identify the responses of fibroblast subpopulations associated with local tissue damage. Finally, spatial omics techniques were introduced to merge spatial transcriptomics with paired scRNA-seq and scATAC-seq datasets for inferring spatial epigenome properties during wound healing as well as mapping the spatial distribution of chromatin accessibility states. By using the aforementioned methodologies, the spatiotemporal dynamic changes occurring in fibroblasts during wound healing were elucidated while simultaneously providing a multi-modal omics framework for future investigations into tissue repair.

Compared with single-cell transcription sequencing technology, the coverage and accuracy of single-cell epigenetic sequencing technology remains relatively low, which is a challenge in epigenetic sequencing technology [104]. In recent years, epigenetic sequencing technologies have been continuously developed that help researchers to decipher epigenetic heterogeneity among rare cell populations and reveal the epigenome of rare cell types, ensuring great progress in the development and application of molecular biology technologies. However, epigenetic sequencing technology has few practical applications for wound healing. Herein, we predict a revolution in the field of wound healing given the rapid development of single-cell epigenetic sequencing.

### Current SCS technology applications in wound healing studies

#### Differences in fetal and adult wound healing

The degree of wound healing is related to the growth and development of the organism. During the process of wound healing in adults, the fibers proliferate rapidly to shield the wound from further damage. However, fetal skin has a more remarkable regenerative ability and healing leaves no scar tissue [105]. Fibroblasts present the main difference in wound repair between adults and fetuses. Rinkevich *et al*. [106] isolated fibroblasts from murine dorsal skin using FACS separation and identified CD26 as a distinguishing marker for different fibroblast types. SCS was used to determine the exact point of time at which scar transformation occurred. scRNA-seq was also used to analyze data on different cell types and specific gene expression patterns in the placenta. By combining mammalian and human placenta SCS data, these studies deepen our understanding of the role of tissue-resident stem cells in mother-to-fetus communication, laying a foundation for further exploration of therapeutic methods in wound healing [107].

#### Skin fibrosis

A keloid is a fibrotic skin lesion characterized by excessive ECM deposition in the wound that is distinct from a hypertrophic scar and is usually the result of surgery, trauma, or burns [108]. Fibroblast proliferation and ECM deposition are critical components of hypertrophic scar (HS) formation. SCS has revealed distinct subpopulations of fibroblasts in HS. Song *et al*. [109] analyzed RNA-seq data from healthy skin and HS samples to identify key gene modules specific to HS fibroblasts using the high-dimensional weighted gene co-expression network analysis method. Gene enrichment analysis highlighted gene involvement in essential processes, such as ECM tissue remodeling, collagen-containing ECM organization, and oxidative phosphorylation. To refine gene selection, seven additional machine-learning methods were used, narrowing the genes down to three: Cathepsin K (*CTSK*), Collagen Triple Helix Repeat Containing 1 (*CTHRC1*), and Thrombospondin-2 (*THBS2*). A comprehensive approach was used to identify biomarkers, with particular emphasis on the role of *THBS2* as a significant factor influencing HS formation and progression via downstream TGF-β1/PSmad2/3 pathway activation. The integration of these methodologies underscores the potential value of SCS analysis combined with multiple machine-learning techniques to identify novel biomarkers associated with HS. Systemic sclerosis (SSc) is also a heterogeneous disease connected with skin fibrosis. Wu *et al*. [110] analyzed the DEG between patients with SSc and healthy volunteers via SCS and found that the Yes-associated protein/Transcriptional co-activator with PDZ-binding motif (YAP/TAZ) pathway was enriched in the fibroblasts of patients with SSc, which was further verified by *in vivo* experiments. YAP/TAZ was identified to inhibit inflammation and fibrosis in mice, demonstrating a potential therapeutic target for SSc. These real-world applications using SCS provide a major medical opportunity to repair abnormal wounds caused by skin fibrosis.

#### Chronic unhealed wounds

Diabetes, vascular disease, and ageing are the main causes of chronic non-healing wounds, leading to diabetic foot ulcers, venous foot ulcers, and pressure ulcers, respectively [111]. As mentioned above, researchers used SCS to comprehensively locate the diabetic wound system and found that multiple fibroblasts were clustered near the wound surface, thus revealing the multifunctional role of fibroblasts in diabetic wound healing and suggesting that fibroblasts may be potential therapeutic targets for diabetic foot ulcers [68, 69]. In addition, angiogenesis is crucial in diabetic wounds, and human dermal microvascular endothelial cells (HDMECs) are an important part of dermal angiogenesis. Du *et al*. [112] isolated HDMECs from diabetic foot ulcers and healthy control skin by cell sorting, identified RAB17 as a potential marker of angiogenesis through scRNA-seq, and as a result found that the angiogenesis capacity of diabetic foot ulcers was related to the dysregulation of RAB17 expression in HDMECs. This discovery provides a potential avenue of wound treatment of diabetic foot ulcers. As mentioned earlier, the skin resembles a separate ecosystem with different kinds of bacteria, viruses, and fungi. Elderly people or people with diabetes or burn injuries may experience the harmful effects of these bacteria. He *et al*. [113] screened CD34+ cells in full-layer skin defect wounds of both ordinary and diabetic mice, followed by scRNA-seq to obtain gene expression data. Seven cell types, including endothelial cells, fibroblasts, KCs, macrophages, T cells, SMCs, and chondroids were identified within the CD34+ cells in the wound tissue of both groups at 4 days post-injury. Notably, fibroblasts were further classified into five subgroups. In diabetic mice, CD34+ endothelial cells, fibroblast subgroup 1, fibroblast subgroup 4, KC, and chondroid cells were increased in the wound tissue; whereas in CD34 + fibroblast subgroup 2 and fibroblast subgroup 3, SMCs were observed. Furthermore, Differential Gene Expression (DEG) analysis and functional enrichment analysis revealed the significant role played by CD34+ cells in wound repair as well as their related mechanisms. This study provides a comprehensive description of the types and functions of CD34+ cells in full-layer skin defect wounds for both ordinary and diabetic mice using the SCS approach, and demonstrates their high heterogeneity during the wound healing process. These findings deepen our understanding regarding the wound healing mechanism while providing a theoretical basis for the clinical application of CD34+ cells in treating diabetic wounds. Yakupu *et al*. [114] analyzed the changes in the epidermal cell–cell communication network during chronic wound healing using scRNA-seq data, and found that compared with acute wounds, the interactions between cells and molecules in chronic wounds such as pressure ulcers were increased, and the main source was the significantly up-regulated PARs signaling pathway of melanocytes. Increased melanocytes in pressure sore wounds can synthesize and secrete cathepsin G and may promote the inflammation of chronic wounds through cathepsin G-F2RL1 (F2R Like Trypsin Receptor 1).

## Summary and outlook

The skin comprises fixed cell populations, and each resident cell subpopulation may have subtypes that preferentially respond to different trauma signals; the interaction between resident cell types and subtypes and how they interact with cells in the wound environment is the focus of wound healing [115]. In this particular study, SCS was concentrated on RNA-seq to investigate the effect of DNA sequencing and epigenetic sequencing on wound healing. SCS technology holds great significance in the field of SCS research; under the premise of the correct use of SCS technology and bioinformatics analysis, information such as tissue cell grouping, differential genes, and enrichment signaling pathways can be obtained. This helps us to understand the disease to a greater extent, further promotes the molecular cognition of the disease, solves the difficulties in cell composition analysis, and provides a new perspective for analyzing the different wound healing mechanisms and exploring new therapeutic methods.

Although the literature on scDNA-seq in wound healing is relatively limited, there are compelling reasons to be optimistic about the future of DNA sequencing in this field. The applications of DNA sequencing have been significantly expanding over the past few decades. The ultimate objective of single-cell genome sequencing is to achieve precise detection of CNVs, insertions and deletions, single nucleotide variants, and other structural variants, thereby providing accurate genetic information for personalized medicine and disease treatment. Numerous laboratories are continuously enhancing single-cell amplification methods [116] to improve fidelity and reduce bias, which will help overcome the challenges of current techniques. With scientists dedicating intensive efforts in this area, significant advancements in single-cell genome sequencing technology are expected in the near future. This will enable us to gain an understanding of how genetic differences between individuals impact health and disease development while offering reliable data for precision medicine and personalized wound treatment.

In recent years, SCS technology development has brought revolutionary changes to the biomedical field. In wound healing, the application of SCS technology has also made major breakthroughs. Traditionally, the various cell types and signaling pathways involved in wound healing have been relatively poorly understood. However, with the advent of SCS technology, researchers can deeply analyze the gene and protein information expressed by each cell at different points in time, revealing the complex and diverse cell subsets and functional characteristics of the wound healing process. Through SCS technology, some key regulatory factors and signaling pathways that were previously overlooked or unknown in the area around the wound were discovered. These new findings contribute to a comprehensive understanding of the mechanisms of interaction between different cell types during wound healing and provide new ideas for the treatment of related diseases. In addition, SCS technology can also be used to achieve personalized treatment programs. By analyzing the expression levels of various key genes in different types of cells and the quantities and states in patient tissues, treatment programs can be tailored according to individual differences to improve the effectivity of treatment and reduce side effects.

However, this research area remains in the preliminary stages, and we should be aware of the limitations of SCS. For example, instant sequencing cannot completely decipher the transcription map, since transcription occurs as a pulse, with the frequency, magnitude, and extent of these pulses varying with chromosome location. Additionally, the frequency, magnitude, and extent of transcription bursts vary with chromosome location; therefore, instant sequencing cannot completely decipher the transcription map [117]. Further, scRNA-seq alone can only identify different spatial distribution patterns of RNA associated with encoded protein features and cannot link genotypes to phenotypes [118]. Moreover, SCS technology has reduced sensitivity to low-expression genes and increased error owing to low material content, technical noise, and low throughput. In the future, the relevant mechanisms should be studied using powerful and reliable genetic data analysis tools that can analyze the SCS data. In addition, the time and cost of this technology should be reduced as much as possible, and effective methods for clinical practice should be applied. In addition to cost, different types of RNA-seq experiments necessitate varying sequencing read lengths and depths (the number of reads per sample). Specifically, the evaluation of RNA-seq primarily relies on read depth rather than coverage. To detect low-expression genes, it is imperative to increase the read depth. The choice of reading depth in an RNA-seq study depends on its specific target. Most experiments require 5–200 million reads per sample, contingent upon the complexity and size of the organism. Although the length of reads is determined by the particular application and library size, a sequencing read length exceeding that of the insert will not yield additional valuable data. If the field can overcome these limitations, SCS will bring about key advances in the understanding of skin wound healing and the practical treatment of skin wounds.

## Conclusions

SCS technology has achieved significant advancements regarding its application in wound healing. First, by conducting gene expression analysis on various types of individual cells in the tissue surrounding the wound, we uncover the expression patterns of key regulatory genes during different healing stages, providing a solid theoretical foundation for treatment strategies. Second, comparing the differential gene expression profiles of specific cell types between normal skin and injured tissues enables the identification of potential biomarkers closely associated with wound repair, presenting valuable clinical application. Lastly, integrating SCS results into clinical practice enables personalized treatment design. Detailed analysis at the single-cell level using tissue samples from the trauma site of a specific patient enables accurate assessment of prognostic risk and facilitates an optimized drug administration plan with reduced dosages and minimized side effects. In conclusion, SCS technology presents substantial application in the field of wound healing and is poised to drive further breakthroughs in related domains. Furthermore, SCS will likely be widely adopted in clinical settings, ultimately benefiting patients by enhancing their access to cutting-edge scientific findings.
